# Editorials for ‘Advances in Cold Plasma in Biomedicines’

**DOI:** 10.3390/biomedicines10112731

**Published:** 2022-10-28

**Authors:** Gyoo-Cheon Kim

**Affiliations:** Department of Oral Anatomy and Cell Biology, School of Dentistry, Pusan National University, Yangsan 50612, Korea; ki91000m@pusan.ac.kr

## 1. Introduction

Research in the field of plasma medicine has provided many explanations for various phenomena, as well as the involvement of the chemical elements of plasma; however, it still lacks in biological mechanism analyses. In this Special Issue, we called for the identification of mechanisms for biological phenomena induced by cold atmospheric plasma (CAP) to compensate for these problems. Of particular note is that while various journals have previously covered Special Issues concerning plasma medicine, “*Biomedicines*” is believed to be the first medical journal to have promoted a Special Issue thereof.

A total of 10 papers was published in this Special Issue, of which 2 were review articles and the other 8 research articles; they could be classified as being very high-quality papers, mostly analyzing tissues beyond the cellular level. The topics covered in each paper also varied from anticancer effects to the expression of regenerative factors, from wound healing to the inhibition of dermatosclerosis and differentiation of osteoblast cells, and the review paper also mentioned recent research trends in plasma medicine and the basic concepts allowing plasma scientists to better understand current biological knowledge.

This Editorial briefly introduces the articles comprising this Special Issue.

## 2. Two Review Articles Introducing the Role of the Direct Treatment of Cold Plasma and Plasma-Activated Liquid in the Medical Field

One of cold plasma’s most known biological properties is its capacity to have a destructive effect on several biological targets. The review article by Yan et al. described the fact that CAP can be used for treating cancer, able to destroy various types of cancer cells by inducing apoptosis, autophagy-associated cell death or necrotic cell death, depending on the plasma treatment properties [1]. By introducing several studies testing CAP’s anticancer effect, its possible mechanisms were suggested as well. In addition, they introduced studies that verified how CAP’s destructive effect effectively removed viruses as well as various types of bacteria. The authors also introduced the fact that, among CAP’s various working elements, not only chemical elements (ROS, RNS, ions, etc.) but also physical elements (electromagnetic elements, light and heat) play an important role in the destructive effect of CAP.

Meanwhile, in their great review, Kim et al. introduced the beneficial role of plasma-activated liquid (PAL) in the medical field [2]. Although a direct treatment using CAP can be very powerful, its use is limited due to its restricted tissue-penetrating ability. In the medical field, PAL usage can be expanded for accessing body parts where a direct application of plasma would be difficult. This review described the chemical elements of PAL and how to generate it, as well as introducing its beneficial roles in sterilization, disinfection, tissue regeneration and cancer treatment. Furthermore, it highlighted PAL’s role in the activation of various solutions that can then be used for medical purposes. Due to the safety of PAL being confirmed using short-term tests on a mouse model, a long-term evaluation using animal models would be needed before the medical use of PAL can be approved.

## 3. Articles Introducing the Anticancer Effect of Direct Treatment with Cold Plasma

Several efforts have been contributed to directly treating cancer with the use of CAP. In this Special Issue, Nitsch et al. tested the effect of an argon plasma jet device (kINPen med) on two chondrosarcoma cell lines, W 1353 and CAL 78 [3]. According to the study, the CAP treatment reduced cell viability, migration and metabolism. Furthermore, it was proven that CAP induced apoptotic cell death in both cells. Although this study was conducted in an in vitro system only, the results suggested that CAP could have considerable treatment benefits, considering that surgery is the only treatment option for chondrosarcoma. The research article by Choi et al. suggested novel approaches for using CAP in combination with gold nanoparticles to treat oral squamous cell carcinoma (OSCC) [4]. According to the research therein, they applied a no-ozone cold plasm to SCC25 and HaCaT cell lines in combination with gold nanoparticles conjugated with the antibody-targeting p-FAK protein (p-FAK/GNP); this combinational treatment successfully triggered OSCC-specific immediate cell death. They suggested that CAP’s charged particles promoted the surface plasmon resonance activity of gold nanoparticles, so that it could induce the immediate cell death of OSCC. Furthermore, the strong anti-OSCC activity of the combinational use of no-ozone cold plasma and p-FAK/GNP was confirmed in an OSCC xenograft mouse model; its effect was much more powerful than merely using CAP on its own. Based on their results, the authors suggested that the combinational treatment of CAP and p-FAK/GNP can be a novel treatment for OSCC.

## 4. Possible Role of Plasma-Activated Liquids for Treating Cancer

As a review by Kim et al. in this Special Issue introduced, there have been many studies conducted elucidating the beneficial role of PAL for treating cancer. The research article by Kong et al. described the anticancer effects of PAL using three tumor animal models [5]. In this study, saline was subjected to a treatment that used a device to generate plasma using ambient air. Since the plasma treatment decreased the pH of the saline, the effect of the plasma-activated saline (PAS) was compared to acidified saline. In the results of the experiments in the xenograft model using A375 melanoma cells, the tumor size was significantly reduced with the PAS injection. On the basis of an ultra-high-performance liquid tandem chromatography quadrupole time-of-flight mass spectrometry analysis of the tumor cell metabolism, the authors insisted that the glycerophospholipid metabolic pathway was the most susceptible metabolic pathway for the PAS-mediated antimelanoma activity. The anticancer activity of PAS was further confirmed in xenograft mouse models for OSCC and non-small-cell lung cancer. The authors also confirmed that the long term use of PAS had few side effects in the three animal models, and suggested that PAL could serve as a potential therapeutic approach for cancer treatment in the near future.

Meanwhile, in a research article by Brito et al., the possible role of PAL for treating peritoneal carcinomatosis using the Ehrlich Ascites carcinoma (EAC) model [6] was suggested. Since EAC mainly grows as a suspension in the peritoneal cavity of mice, this model was suited to test PAL’s anticancer activity. In this article, plasma-oxidized saline (POS) was produced by treating the saline with an argon plasma jet, kINPen med. The five rounds of POS injections led to a reduction in a tumor due to the modulation of EAC cell growth and metabolic activity. Furthermore, the POS injection promoted a decrease in the antioxidant capacity of tumor cells and an increase in lipid oxidation in the ascites, while there were no side effects observed. The authors suggested that the POS is a promising candidate for targeting peritoneal carcinomatosis and EAC is a convenient model for analyzing innovative POS approaches.

## 5. Articles Introducing the Medical Effects of Cold Plasma Other Than Anticancer Efficacy

In addition to CAP’s anticancer effect, there are lots of studies elucidating its beneficial role in several human diseases. In the article by Arndt et al., CAP’s beneficial role for treating localized scleroderma was tested using in vitro and in vivo models [7]. The device they used was an argon-based plasma-generating device, the MicroPlaSterβ^®^. In this article, although the direct CAP treatment used for human localized scleroderma-derived fibroblasts (hLSFs) failed to reduce fibrotic markers such as collagen type I and alpha smooth muscle actin, cell motility was significantly reduced through the induction of metalloproteinase 1. Furthermore, the CAP hLSF treatment significantly reduced the expression of proinflammatory cytokines. The authors confirmed the anti-fibrotic effect of CAP through a test using bleomycin-induced dermal fibrosis, and suggested that the use of CAP could be an option for treating localized scleroderma.

Several researchers explored various studies to validate the beneficial role of CAP in wounds. In this Special Issue a research article by Choi et al. tested the effect of helium-based CAP in diabetic wounds infected with Candida albicans using a mouse model [8]. The results of this study showed that the CAP treatment not only reduced the fungal infection, but also accelerated the process of wound healing. Since diabetes mellitus leaves patients susceptible to chronic wounds and various infections, including fungal ones, this fascinating study provides clues to the usefulness of cap for diabetic wounds.

There are many reports elucidating the beneficial roles of CAP in the oral cavity. Two research articles in this Special Issue introduced CAP’s oral-tissue-regenerative activity. The study by Eggers et al. tested the effect of an argon-based plasma jet, kINPen med, on CAP’s tissue-regenerative activity using human gingival fibroblasts, keratinocytes and human gingival biopsies [9]. In this study, a 30 s CAP treatment led to an increase in wound-healing-related genes and proteins, such as ki-67 and MMP1, whereas a treatment for more than 60 sec induced apoptotic gene expression in cells and superficial damage to the epithelium. Based on their results, the authors suggested that CAP used for a brief amount of time after oral surgery could help with wound healing. The study by Choi et al. reported on CAP’s possible role in bone regeneration in the oral cavity [10]. In this study, they used argon-based no-ozone cold plasma to treat periodontal ligament cells and investigated its effect on osteoblastic differentiation and bone formation. Their results clearly showed that the CAP treatment induced osteoblastic differentiation by promoting the expression of osteoblast differentiation-promoting genes (alkaline phosphatases, osteocalcin, osteonectin and osteopontin) and the activation of alkaline phosphatase. Since cell differentiation capable of differentiating into osteoblasts is important for the recovery of periodontitis patients, CAP could be a good treatment.

## 6. Conclusions and Future Perspectives

Studies are underway to validate the novel medical efficacy of CAP and to uncover its specific mechanism of action. In this Special Issue, the papers not only reported on CAP’s strong bactericidal, wound-healing, tissue-regenerative and anticancer effects, but also its mechanism. In addition, various studies were conducted to develop ways to increase the medical efficacy of CAP to diseases.

Techniques using CAP could be the new answer to diseases that have been difficult to treat in the past, providing a much better quality of life than what is available now. Recently, several startups produced medical devices using CAP. After the process of obtaining medical device approval in each country, this is expected to create a novel medical device market.
